# Medical Clerkship in a State Registration and Reception Center for Forced Migrants in Germany: Students’ Experiences, Teachable Moments, and Psychological Burden

**DOI:** 10.3390/ijerph16101704

**Published:** 2019-05-15

**Authors:** David Kindermann, Carolin Schmid, Cassandra Derreza-Greeven, Florian Junne, Hans-Christoph Friederich, Christoph Nikendei

**Affiliations:** 1 Department of General Internal Medicine and Psychosomatics, University Hospital Heidelberg, University of Heidelberg, 69115 Heidelberg, Germany; carolin_schmid@gmx.de (C.S.); Cassandra.Derreza-Greeven@med.uni-heidelberg.de (C.D.-G.); Hans-Christoph.Friederich@med.uni-heidelberg.de (H.-C.F.); Christoph.Nikendei@med.uni-heidelberg.de (C.N.); 2 Department of Psychosomatic Medicine and Psychotherapy, University Hospital Tübingen, University of Tübingen, 72076 Tübingen, Germany; Florian.Junne@med.uni-tuebingen.de

**Keywords:** global health, medical clerkship, refugees, secondary traumatization, teachable moments, medical curriculum

## Abstract

Aspects of global health are becoming increasingly relevant for doctors of future generations. However, medical curricula rarely include courses which focus on global health or forced migration. Furthermore, it remains unclear whether students are at risk to develop psychological strain, after being confronted with highly burdened or even traumatized asylum seekers. This is a prospective study using a mixed-methods approach. We included *n* = 22 medical students performing a medical clerkship in a state registration and reception center for refugees. By applying (1) qualitative interviews, (2) reflective diaries, and (3) psychometric questionnaires, we examined the students’ experiences, teachable moments, and potential psychological burdens. In the interviews, the students emphasized the importance of cultural sensitivity during their clerkship. However, they also reported cognitive changes concerning their views of themselves and the world in general; this could indicate vicarious traumatization. The reflective diaries displayed high learning achievements. According to the psychometric questionnaires, the assignment in the reception center had not caused any significant psychological strain for the students. By completing their medical clerkship in a reception center, students were able to improve their medical, organizational, and interactional knowledge and skills. Furthermore, they reported that they had broadened their personal and cultural horizons.

## 1. Introduction

In 2017, nearly 200,000 refugees arrived in Germany and applied for asylum to find protection from prosecution, violence, and war [1]; the most common countries of origin were Syria, Iraq, and Afghanistan. More than 9000 of these refugees were unaccompanied minors [1]. Forced migrants have a high prevalence of psychological strain due to the awful experiences in their home countries or during their flight: Manok et al. [2] assessed asylum seekers attending a psychosocial outpatient clinic in the state registration and reception center in southwestern Germany, in which the present study was also conducted. They found prevalence rates of 38% for post-traumatic stress disorder (PTSD), 27% for depressive disorders, and 17% for substance abuse [2]. Moreover, in a recent systematic review, Bozorgmehr et al. [3] indicated that the prevalence of PTSD in forced migrants ranged between 6.7% and 76% depending on the means of evaluation and whether institutional or organizational study samples were assessed. In comparison, the one-month prevalence of PTSD in a Western norm sample was 2.3% [4]. In addition to the reported mental health problems, several studies have also indicated higher rates of infectious diseases in refugees [5,6]. In particular, a high prevalence of tuberculosis [7] and malaria [8] was discovered. Therefore, we assume that forced migrants are a highly burdened population, suffering from severe mental as well as physical disorders. However, it is complex and challenging for the doctors in charge to offer adequate treatment to forced migrants because there are clinical, psychological, intercultural, and linguistic barriers to overcome.

Globalization increasingly plays a role in humans’ daily lives in general and in medical healthcare in particular. Thus, “Global Health” will become increasingly important for future generations of doctors [9]. Despite the growing demand for global health education, medical curricula rarely include issues concerning global health [10,11]. In 2013, Peluso et al. [12] investigated the prevalence of structured, longitudinal global health programs in US medical schools. Only 24% of the examined institutions offered such programs and little standardization was observed [12]. Furthermore, consensus on the educational content [11] and required competencies which medical students should acquire in mandatory or elective global health courses is lacking [13]. In a literature review from 2010, Battat et al. [13] identified competencies most frequently deemed relevant for global health education: these comprise an understanding of the global burden of diseases, travel medicine, health care disparities between countries, immigrant health, primary care within diverse cultural settings, and soft intercultural competence skills [13,14]. Peluso et al. [12] (2012), on the other hand, proposed cultural humility, respect, and curiosity towards other cultures to be the most important competency for medical students to learn [15]. In a nutshell, global health programs in medical curricula are still rare and vary greatly in content and didactic approach; although, experiential learning is recommended [13]. Among the scarcely offered courses on global health, classes which concentrate on asylum seekers’ health care provision are even less frequent [10]. The existing programs differ widely in structure and content: i.e., primary care classes, in which students perform a supervised anamnesis and a basic physical screening of refugees [16]; “refugee health nights” for refugees, students, and family physicians to get together and discuss health-related aspects [17]; and simulated situations for educational purposes, for example, teaching students how to deal with infectious diseases in a refugee camp [18]. Apart from this, global health aspects are often addressed in elective courses which comprise both theoretical and practical issues. For instance, Bateman [10] described an elective course on global health at the Karolinska Institute in Stockholm, Sweden. The course consists of a three-week theoretical part focusing on socioeconomic and cultural aspects, followed by a two-week practical assignment in Tanzania, India or Cuba [10]. However, to the best of our knowledge, there is still a lack of reports on what motivates students to work in global healthcare, what kind of experiences they have, and in which roles they perceive themselves. Furthermore, there is little known about the students’ learning success in this setting.

In registration and reception centers, forced migrants have personal contact with different professional groups, including medical doctors, interpreters, or security personnel. In the context of the asylum procedure or medical examinations, professionals are directly or indirectly confronted with refugees’ personal histories. These often include distressing and traumatic biographical experiences and may result in the staff showing signs of psychological strain [19,20]. Eventually, this may even lead to “secondary traumatic stress” (STS), which is the result of a process called “secondary traumatization” [21,22]. The phenomenon of secondary traumatization describes how symptoms of post-traumatic stress, e.g., intrusions, avoidance, or hyperarousal, are transferred from a primarily traumatized individual to an initially healthy individual [22,23]; this may happen when individuals listen to narratives of traumatic experiences. In a recent cross-sectional study which investigated the psychological burden in interpreters at a state registration and reception center, we found that 21% of the participants fulfilled all criteria of secondary traumatization [20]. Furthermore, there is evidence that medical students may also suffer from STS in the aftermath of a voluntary placement in a reception center (Kindermann et al.; unpublished data). Another concept, which refers to the consequences of being exposed to traumatic narratives, is “vicarious traumatization” (VT). This means that due to the confrontation with traumatic narratives, individuals may experience changes in cognitive schemas concerning the self, others, and the world in general [23,24]. Consequently, basic needs for safety, trust, esteem, intimacy, and control could be disrupted [25].

Since 2016, medical students have the opportunity to conduct part of their obligatory medical clerkships in the state registration and reception center for forced migrants in Heidelberg–Kirchheim. In the present study, we applied a mixed-methods approach: we used (1) qualitative interviews to analyze the students’ motivations and experiences, (2) reflective diaries to explore the students’ learning achievements, and (3) psychometric questionnaires to examine psychological strain, in terms of depressive symptoms, anxiety, mental and physical wellbeing, and potential secondary traumatization in the participants. The study aimed to analyze a new learning context, connecting aspects of global health with health care provision of refugees as well as focusing on psychological burden in the students.

## 2. Materials and Methods

### 2.1. Study Design

Following the classification of mixed-methods approaches by Creswell and Clarke [26,27], we applied a concurrent triangulated design. We conducted a prospective study comprising (1) qualitative interviews with a pre–post design, (2) reflective diaries which were filled in by the participants during their medical clerkship, and (3) psychometric questionnaires with a pre–post design. The study aimed to investigate (a) medical students’ motives, expectations, and personal experiences in the refugee reception center, (b) their general learning progress, the accomplishment of learning goals, the students’ perceived role, and (c) their psychological burden in terms of symptoms of depression, anxiety, mental, and physical wellbeing, and secondary traumatization. For this purpose, *n* = 22 medical students of the University of Heidelberg who did their medical clerkship in the outpatient clinic of the Medical Treatment Center of the Heidelberg–Kirchheim State registration and reception center “Patrick Henry Village” (PHV) participated in the study. The qualitative and quantitative results of the study were equally weighted and examined for possible convergence [26].

### 2.2. The Medical Clerkship

In German medical curricula, students have to complete four months of medical clerkships (“Famulatur”) during their semester breaks between the 4th and the 10th semester [28]. These medical clerkships are obligatory for students in order to be admitted to the final state examination to become a medical doctor. These four months can be divided into different time intervals, with a minimum of 14 days comprising one interval. The students can either complete their medical clerkships in inpatient settings or conduct a period of up to four weeks in an outpatient setting. The medical specialization in which the students perform their internships can be chosen freely.

### 2.3. Student Sample

In our sample, we included all medical students who did their medical clerkship at the outpatient clinic in PHV between June 2016 and November 2017 and agreed to participate. The participant recruitment for the pre-interviews took place before medical students began working in the center between May 2016 and October 2017. The medical students filled in the reflective diaries throughout their placements. We administered the psychometric questionnaires before the students had started and after they had completed their medical clerkships between May 2016 and November 2017. The post-interviews were conducted between June 2016 and December 2017. The inclusion criterion for the post-interviews was that students had completed a clerkship with a minimum of 14 days in the outpatient clinic of the PHV.

### 2.4. The State Registration and Reception Center “Patrick Henry Village” in Heidelberg–Kirchheim

Late in 2014, the former US army barracks called “Patrick Henry Village” (PHV) in Heidelberg–Kirchheim, Germany, were converted into an emergency winter accommodation for refugees. In September 2015, the PHV became an official state registration and reception center for forced migrants in the German Federal state of Baden–Württemberg. On an area covering over 90 hectares, this center provides shelter for approximately 6000 asylum seekers. Here, refugees can seek medical help in a provisional medical outpatient clinic for general medicine, pediatrics, gynecology, and psychosocial medicine [2,29]. The clinic is run by physicians registered in Heidelberg and physicians from the University Hospital Heidelberg. In 2017, Manok et al. [2] investigated the PHV sheltered refugees’ sociodemographic data in the context of the PHV’s psychosocial outpatient clinic [2]. The average age of outpatients was 31.18 years (±10.41 years). A total of 35.0% of the patients were female and 65.0% male. Most refugees originally came from Sub-Saharan Africa (33.1%, especially Gambia and Nigeria), Southeast European countries (33.1%, mainly the Balkans, Turkey, and the Caucasus), the Middle East (23.7%, especially Afghanistan and Iraq), and Maghreb countries (9.1%). The most frequently spoken languages were Arabic (10.1%), Serbo-Croatian (9.1%), and Persian (7.6%). The most frequently cited religions were Islam (39.7%) and Christianity (13.6%) [2].

### 2.5. Ethics

Ethical approval was granted by the ethics committee of the University of Heidelberg (Nr. S-694/2015). Study participation was voluntary, and all candidates were assured of confidentiality. The collected personal data was fully pseudonymized. Non-participation had no impact on the course of the clinical placement or university exams. The study was conducted in accordance with the most recent version of the Declaration of Helsinki [30]. We obtained written consent from all participants.

### 2.6. Sociodemographic Data of the Student Sample

Prior to the pre-interviews and the psychometric questionnaires, all participating medical students completed questionnaires assessing sociodemographic information and previous experience in foreign aid. We evaluated group characteristics, such as age, sex, nationality, previous training in a healthcare-related or medical profession, experiences of voluntary work and development aid, and the students’ aspirations concerning their future medical career (see Table 1).

### 2.7. Qualitative Assessment: Semi-Standardized Pre–Post Interviews

We developed the interview questions based on an in-depth literature review as well as discussions in a team of experts (*n* = 3, all of whom were experienced in qualitative research). The interview manual was constructed in a semi-standardized manner [31,32,33,34]. We developed leading, open-ended questions concentrating on the students’ motivations, expectations, experiences, and reflections regarding their medical clerkship in the PHV. If necessary, these questions were followed by encouraging and clarifying questions. In line with the “consolidated criteria for reporting qualitative research”-checklist (COREQ) [35], we informed all participants about the study’s background, objectives, and procedure. The interviews were conducted according to the semi-standardized interview manual in individual, face-to-face settings and were audiotaped. The interviewer was supervised by experienced colleagues.

### 2.8. Qualitative Content Analysis

After verbatim transcription of the resulting audio files (17 pre-interviews and 17 post-interviews), we performed a qualitative content analysis following the principles of inductive category development [36]. First, we conducted a line-by-line open coding of the 34 interview transcriptions. In detail, we identified a single sentence or a passage as a code, representing the most elemental unit of meaning [37]. Using the software “MAXQDA” [38,39], we grouped several codes into individual themes for each participant. Next, we compared the themes between the participants and adapted them, until we could define a number of relevant themes for all participants. The assignment of the respective codes to specific themes was firstly carried out by two analyzers independently, who subsequently discussed their progress to reach consensus (investigator triangulation). Finally, the resulting themes were consolidated into relevant categories.

### 2.9. Reflective Diaries

To evaluate the students’ learning experiences more specifically, we developed a reflective diary based on an in-depth literature review. The diary consisted of 10 pages, with each page corresponding to a single day. The students were instructed to complete one page per day during their medical clerkship. Every page was roughly subdivided into 3 sections: “What was my learning highlight today?”, “Short characterization of the patient”, and “My take-home message for today”. In the first and the third section, students could freely formulate their answers. The second section included detailed questions about which part of the outpatient clinic the students had been assigned to (general medicine, pediatrics, gynecology, or psychosocial medicine), sociodemographic facts on the encountered patient, the main diagnoses in the different outpatient clinics, the student´s function, and their practical involvement during the consultation.

### 2.10. Evaluation of Reflective Diaries

The reflective diaries were analyzed by two experienced investigators who were supervised by an additional investigator. We obtained information regarding the patients, such as age, sex, homeland, the reason for the medical consultation, diagnosis, and therapy. Regarding the students’ reflections on their learning experiences, we applied Bloom´s taxonomy and the Canadian Medical Education Directives for Specialists (CanMEDS) Role Model and assigned the main statements to different categories of these concepts: each reflective diary was firstly analyzed by two experienced investigators independently, assigning (a) the described learning experiences to the respective learning levels according to Bloom’s taxonomy and (b) the medical students’ described activities and perceptions to the respective roles according to the CanMEDS framework. Each learning experience and perceived role was assigned to a maximum of two categories. In a second step, the individual assignments of the two investigators were compared and discussed in an expert group for each reflective diary.

### 2.11. Bloom´s Taxonomy and the CanMEDS Framework

There are two concepts to define and specify the learning experiences of medical students and doctors: Bloom´s taxonomy [40,41] and the CanMEDS framework [42]. The revised version of Bloom´s taxonomy [41] sorts individual learning achievements according to six hierarchical steps. The lowest step is “remembering” something; the act of “evaluating” new knowledge, and eventually, “creating” own ideas are ranked highest. The CanMEDS framework was developed to describe different roles a doctor can take during interactions with patients. In current literature, the CanMEDS model encompasses six different roles: the professional, the communicator, the collaborator, the manager, the health advocate, and the scholar [42]. Both Bloom’s taxonomy and the CanMEDS paradigm can be used to analyze and characterize learning experiences of medical students.

### 2.12. Quantitative Pre–Post Assessment of Psychological Strain

To assess depressive symptoms, we applied the German version of the PHQ-9 depression module [43] of the Patient Health Questionnaire (PHQ) [44]. The PHQ-9 depression module was designed to assess depressive symptoms, the severity of the disorder, and the development of symptoms according to the Diagnostic and Statistical Manual of Mental Disorders, Fifth Edition (DSM-5) [45]. The questionnaire’s validity is very good [43,46,47]. The nine items assess whether a patient has experienced depressive symptoms in the past two weeks. The sum score ranges from 0 (no depressive symptoms) to 27 (all symptoms occur daily). The recommended cut-off score to distinguish between clinical and non-clinical populations is 10 or above. Furthermore, the questionnaire distinguishes between different levels of the severity of depression: minimal symptoms (sum score 0–4), mild symptoms (5–9), moderate symptoms (10–14), and severe symptoms of depression (15–27) [43].

In order to asses symptoms of anxiety, we applied the Generalized Anxiety Disorder Seven-Item Scale (GAD-7) [48]. This questionnaire aims to investigate the presence of anxiety according to DSM-IV criteria over the past two weeks. Items are scored on a scale from 0 (not at all) to 3 (nearly every day). A score of 10 or higher is interpreted as a sign of significant anxiety, scores above 15 point to severe anxiety [48,49]. Like the PHQ-9 depression module, the GAD-7 questionnaire distinguishes different levels of symptom severity: minimal symptoms (sum score 0–4), mild symptoms (5–9), moderate symptoms (10–14), and severe symptoms of anxiety (15–21) [48].

To evaluate the health-related quality of life, we used the SF-12 [50] which is a short form of the worldwide established Short Form Health Survey (SF-36) [51,52]. The SF-12 encompasses 12 items from the SF-36 regarding their relative efficiency or psychometric performance in eight health-related areas (e.g., pain, vitality, psychological functioning). The SF-12 has two resulting scores: one score for physical health and one score for mental health. There is a large German norm sample for the SF-36 [53], which can be administered for the interpretation of the SF-12 [54].

We investigated the occurrence of secondary traumatization by using the German Questionnaire for Secondary Traumatization (FST, Fragebogen für Sekundäre Traumatisierung) [55,56]. The questionnaire comprises 31 items covering the four symptom clusters of PTSD according to the DSM-V. Additionally, the FST includes an individual’s sense of threat and safety behavior. In the authors’ sample, individuals scoring between 65 and 82 points were classified to suffer from moderate secondary traumatization, while participants scoring above 82 were classified to suffer from severe secondary traumatization [56]. In the present study, we asked the participating medical students to rate how often one or more of the 31 symptoms had occurred during the worst week of their assignment on a 5-point Likert scale (1 = never to 5 = very often).

### 2.13. Quantitative Statistical Analysis

The results are presented either as mean values ± standard deviation (SD) or as medians. In order to describe the sample in more detail, we calculated descriptive statistics. We used parametric tests to analyze normally distributed data. A *p*-value < 0.05 was considered as statistically significant. For our statistical analysis, we used the software package IBM^®^ SPSS^®^ Statistics Version 22. A paired samples *t*-test was used for pre–post comparisons. The Student’s *t*-test for independent samples was applied for group comparisons.

## 3. Results

### 3.1. Sample Description and Response Rate

The sociodemographic characteristics of the sample are displayed in Table 1. From a total of *n* = 25 medical students, *n* = 22 students participated in the study. This is a response rate of 88.8%. Interviews were performed on a subsample of *n* = 17 students before, and *n* = 17 students after the medical clerkship. This is a response rate of 77.2%.

### 3.2. Qualitative Assessment: Results of Pre–Post Interviews

The qualitative analysis of the interview transcripts led to 185 single codes from the pre-interviews and 358 single codes from the post-interviews. With reference to these codes, we derived two main categories for the pre-interviews and two main categories for the post-interviews. The main categories of the pre-interviews were: (A) expectations of the medical clerkship and (B) motivation for the assignment. Each of the main categories (A and B) contained 2–6 themes, which were further differentiated into subthemes. The main categories of the post-interviews were: (C) subjective experiences during the medical clerkship and (D) subjective experiences after the medical clerkship and reflection. Each of the main categories (C and D) contained 2–3 themes, which were further differentiated into subthemes. In Table A1 (see Appendix A), we have listed quotes taken from the interviews for each subtheme.

#### 3.2.1. Main Categories and Themes from the Pre-Interviews

(A) Expectations of the medical clerkship *(110 codes)*

The medical students expected their medical clerkship in the outpatient clinic of the reception center to be challenging on an interactional, organizational, and medical level. Some students anticipated to be confronted with problems regarding their communication with the refugees. They thought that it could be a problem that they spoke different languages and came from different cultures.

“[I have been thinking about] the language barrier and how it will influence the quality of treatment. Otherwise I am just a little curious if there will be cultural difficulties and if you will try and speak about specific issues, or if you just formulate some issues badly.”

(A1.11)

Furthermore, the students expected to be exposed to individual background stories, especially in the psychosocial outpatient clinic. Some of the students thought that their experiences during the internship would have an impact on their own psychological constitution. With “impact” they meant emotional strain or a potential change regarding their worldview or moral concepts. Some of the participants expected that they would adjust their view on refugees and migration on the one hand and relativize their own standard of living on the other hand.

“I think it [the medical clerkship] can be stressful when you are confronted with different fates and get in touch with the individual patients.”

(A1.2)

However, most of the participants did not worry about suffering from psychological burden in the aftermath of their shifts in the reception center. They stated that giving medical or psychological aid was just a normal part of their daily professional duties. A few respondents were worried whether they would have enough medical knowledge and skills to provide proper treatment for the refugees. In addition, some of the participants worried about getting into contact with contagious diseases, e.g., tuberculosis, or even being infected. They were not sure what kind of hygienic conditions they would find in the reception center.

(B) Motivation for the assignment *(75 codes)*

Almost all the participants hoped that doing their medical clerkship in the reception center and treating patients with rare diseases would improve their theoretical medical knowledge. The students thought that they would be able to give the patients at the outpatient clinic in the reception center more support than the patients they would encounter when conducting a medical clerkship in a typical Western general practice or inpatient clinic. Many participants hoped that they would gain a more personal perspective on refugees and their situations. They thought that this clerkship would give them a better understanding of global health issues in general and the different cultures of the refugees in particular. The students expected that these experiences would be useful for their future careers as doctors.

“I look forward to perhaps broadening my horizon and to interacting with different patients; I can probably use this expertise for my future profession.”

(A1.9)

Aside from this, the majority of participants were interested in learning about the refugees’ personal background stories and what kind of experience they had had during their flight.

#### 3.2.2. Main Categories and Themes from the Post-Interviews

(C) Subjective experiences during the medical clerkship *(209 codes)*

Most of the students had the opportunity to assist medical doctors during the consultations and were able to extend their theoretical medical knowledge and improve their practical skills. For instance, they were allowed to conduct medical examinations, do ultrasound scans, or take blood samples from the patients. The students thought that one of the major challenges in communicating with the asylum seekers was the language barrier.

“In fact, the main problem was the language barrier, which was not really unbreachable but nevertheless a major obstacle, especially when there was no interpreter available. Some common foreign languages were helpful, for instance English or French. Fortunately, I speak a bit of French.”

(A1.2)

Furthermore, the students found it difficult to diagnose and treat psychological conditions and chronic, somatic diseases. They indicated that they had often been confronted directly with the refugees’ individual background stories, e.g., during the anamnesis. Almost every student felt overwhelmed by this.

“There were a few individual background stories about which I had to think again, because those stories were just mad; although you hear about those stories in the newscast, it is crazy if someone who experienced such things on their own is standing right in front of you. Especially in the psychosocial outpatient clinic you hear a lot of those stories. In general medicine you sometimes do not even get in touch with it [the stories], but in Psychosomatics [the psychosocial outpatient clinic] there were some cases which kept coming back to me later on.”

(A1.4)

In this context, participants often reported feeling helpless towards the refugees because they could not do anything to alter their traumatic experiences. The students had encountered cultural differences, especially in regard to how people of different gender interacted with them and how psychological or somatic symptoms were perceived or communicated by the patients. As many of them had expected beforehand, they had to deal with a very diverse spectrum of bodily diseases in comparison to inpatient clinics in Germany. The participants stated that they had encountered many tropical or parasitical diseases, tuberculosis, hepatitis B, scabies, and sexual transmitted diseases. The participating students welcomed the opportunity of gaining medical knowledge about these disorders.

(D) Subjective experiences after the medical clerkship and reflection *(149 codes)*

Despite having had some negative experiences and being exhausted from the assignment, the students gave an overall positive feedback on the medical clerkship at the reception center. In the aftermath, several participants were seriously concerned with some of the refugees’ individual and personal background stories. Above all, they had been confronted with these stories at the psychosocial or the gynecology outpatient clinic. Many students continued to think about the refugees’ reasons to leave their home countries and worried about what would happen to them in the future. Looking back, most students reported feeling helpless. This feeling resulted from not being able to change anything about the traumatic experiences of the asylum seekers. Furthermore, some participants reported changes in their personal views on the world and on themselves; they felt powerless in view of worldwide inequality, war, and forced migration. These alterations can be seen as cognitive changes and may even be a sign of vicarious traumatization (VT).

“In fact, I had the feeling that this problem [war and flight] was so big, that you cannot do a lot about it. It feels like waking up, saying ´Okay, this is a huge problem and I have to do something, but I am just not in the position to do so´. In fact, this is a sad feeling; but actually, it seems to be a mixture of positive and negative feelings.”

(A1.6)

By talking about their assignment with colleagues and family members afterwards, the respondents tried to deal with their negative feelings. Some of the participants realized that the term “refugees” was a very heterogeneous concept and that their assignment at the reception center had enabled them to gain a more specific and differentiated perspective on refugees and their histories. Most of the participants felt empowered in their moral concepts. The medical clerkship had highlighted the importance of meeting all patients in a respectful and unprejudiced way, regardless of their origin. The students felt content to have had the opportunity to help people who were in need and suffered from mental or physical strain. Therefore, they had felt “useful”. The participants thought that they had learned a lot because they were expected to work independently, also regarding rare medical conditions. Some of them believed that the fascinating and exciting aspects of the medical clerkship had increased their knowledge. Finally, the interviewees reported having relativized their own standard of living when comparing their personal lives to the refugees’ living conditions. Overall, many students drew the conclusion that the medical clerkship had been an important opportunity to broaden their personal and cultural horizons.

### 3.3. The Students’ Reflective Diaries

**Figure 1:**
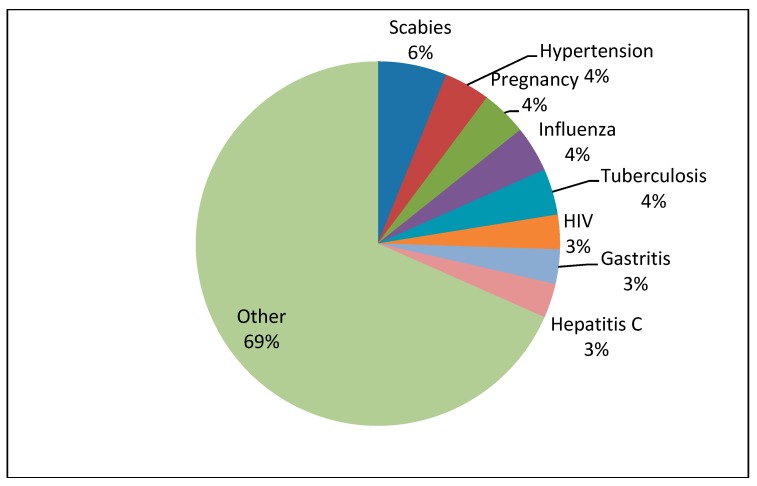
Results from the reflective diaries: showing the proportion of different diagnoses which were made in the outpatient clinic for general medicine.

**Figure 2:**
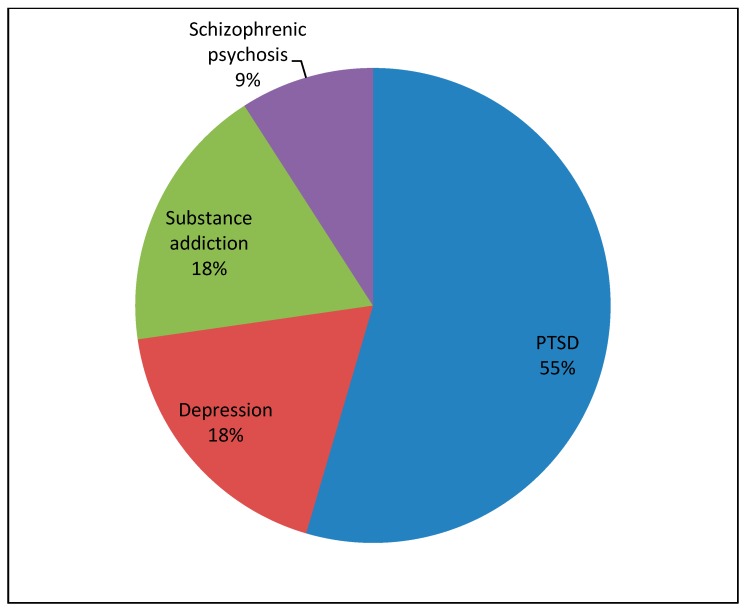
Results from the reflective diaries: showing the proportion of different diagnoses which were made in the outpatient clinic for psychosocial medicine.

**Figure 3:**
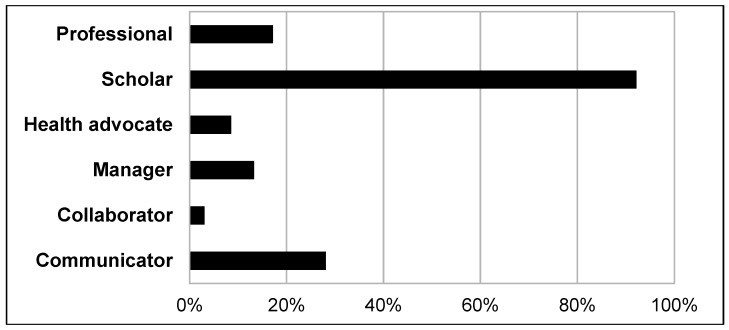
Results from the reflective diaries: distribution and frequency of roles according to the Canadian Medical Education Directives for Specialists (CanMEDS)- concept based on the patient cases as described by the participating students.

**Figure 4:**
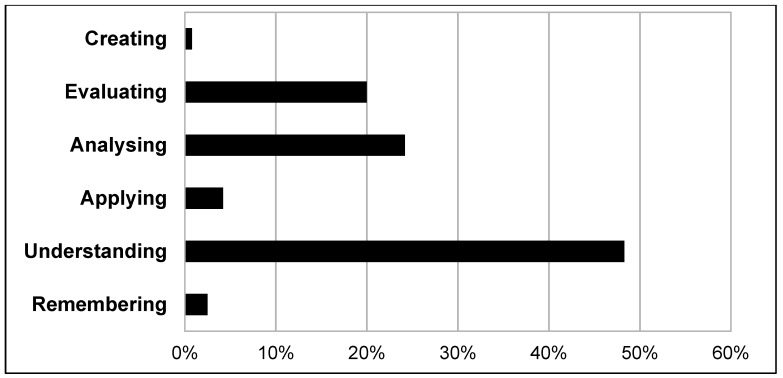
Results from the reflective diaries: distribution and frequency of the different stages of Bloom´s taxonomy based on the patient cases as described by the participating students.

A total of 126 patient cases with corresponding teachable moments were described in the reflective diaries. The medical students documented from 1 up to 10 teachable moments each, resulting in a mean of 5.7 teachable moments per student. Table 2 describes the characteristics of patients who were the basis of the teachable moments commented on in the reflective diaries. The majority of patients, whom the medical students encountered, suffered from general somatic complaints and was treated in the area of general medicine in the outpatient clinic (see Figure 1 and Figure 2). Referring to the CanMEDS Role Model, the participants mostly reported on teachable moments in which they had taken the role of “scholar” or “communicator” (see Figure 3). In the role of scholars, medical students or physicians show a lifelong commitment to reflexive learning and to the creation, application, and transmission of knowledge [42]. Acting in the role of communicators, physicians or medical students form relationships with patients, and if present, with their families facilitating the gathering and sharing of essential information for effective health care [42]. Analyzing the reported teachable moments following Bloom’s taxonomy, the students mostly achieved the learning level of “understanding” in the presented cases. This rank represents the process of constructing meaning by interpreting, exemplifying, classifying, summarizing, inferring, comparing, and explaining bits of information (see Figure 4).

### 3.4. Psychological Strain and Secondary Traumatization of the Medical Students

Table 3 depicts our quantitative results concerning the psychological strain assessed in the medical students. We obtained the following PHQ-9 scores prior to the students’ assignment in the PHV: 64% of the examined participants showed minimal depressive symptoms, 13% displayed mild depressive symptoms, and 4.5% presented moderate symptoms of depression. After having completed their medical clerkship, 64% of the medical students showed minimal depressive symptoms and 18% displayed mild depressive symptoms. The pre–post comparison of the PHQ-9 scores did not yield a significant difference (t(21) = −0.495, *p* = 0.626). Regarding GAD-7 scores prior to the students’ placement in the PHV, our evaluation resulted in the following scores: 77 % of the students displayed minimal symptoms of anxiety and 13% presented mild symptoms of anxiety. After having finished their assignments, 77% of the students had minimal symptoms of anxiety and 9% showed mild symptoms of anxiety. The pre–post comparison of GAD-7 scores did not result in any significant difference (t(21) = 0.160, *p* = 0.875). The SF-12, measuring health-related quality of life, did not uncover a significant difference between pre- and post-scores. This was the case neither for mental (t(20) = 0.567, *p* = 0.577), nor for physical wellbeing (t(20) = −1.473, *p* = 0.156). The results of the FST showed that none of the participants had scores that indicated secondary traumatization in the aftermath of their medical clerkships. We did not uncover any gender differences in the psychometric values.

## 4. Discussion

The present study aimed to analyze the expectations, motivation, experiences, teachable moments, and potential psychological strain of medical students who were doing part of their obligatory medical clerkship in a state registration and reception center for forced migrants. In the interviews, the participants reported a large spectrum of enriching experiences, especially emphasizing that they had gained cultural sensitivity and broadened their personal horizons. However, some students showed possible signs of VT, such as negative changes of cognitive schemas. The teachable moments which the students described in the reflective diaries were mostly based on their roles as scholars or communicators and mostly reached Bloom’s taxonomic level of understanding and analyzing. Taken together with the qualitative data, this indicates that their knowledge increased in organizational, interactional, medical, and intercultural aspects. Concerning the assessment of psychological strain in terms of depressive symptoms, anxiety, and general wellbeing, the pre–post comparison did not yield a significant difference. Further, the comparison of our sample of students to a representative norm sample did not show any significant differences, either. According to the FST questionnaire, none of the participants displayed signs of STS after their placement. Our results lead to the conclusion that more courses and internships with the focus on health care for refugees and aspects of global health should be included in medical curricula, in order to meet the demands of a progressing globalization. In our discussion we would like to highlight how we integrated the results from the triangulated mixed-methods approach [26]. Therefore, we have structured the discussion in a topic-centered manner.

### 4.1. Psychological Distress and STS

Doing an internship in a state registration and reception center often brings medical students into contact with tropical or parasitic diseases, some of which they would not encounter in other clinical settings in Western countries. In the pre-interviews, some participants were worried that they would have to deal with a lot of very contagious diseases. At the same time, they were not sure whether the hygienic conditions in the reception center would be sufficient. These worries are in line with fears in the general population: a lot of people are afraid of the outbreak of infectious diseases brought to Europe by refugees—although the actual risk is estimated to be very low [58,59,60]. With regard to medical clerkships in the context of health care for refugees, this observation highlights how important it is to provide students with enough information about possible risks and existing conditions before they start with their placement. A first step could be to give introductory courses.

In the post-interviews, several medical students stated that it had been a great challenge to deal with patients suffering from psychological diseases, mostly PTSD. In this regard, some of the participants reported intense feelings of helplessness because they were not able to “change” the personal fate of the refugees. Previous studies on trauma therapy have assessed similar feelings of helplessness, which were also induced by listening to traumatic narratives. It has been established that this confrontation with trauma represents an extreme psychological burden for therapists [23,25,61]. Moreover, the symptoms of the primarily traumatized individual can be “transferred” to the listener. This “symptomatic transfer” is probably generated through an empathic process: the act of merely listening to a story may evoke vivid mental imagery and could result in psychological burden or even secondary traumatic stress (STS) [62,63]. In the present study, the psychometric assessment with the FST showed that none of the medical students presented any signs of STS in the aftermath of their medical clerkship. The mean scores of the FST were not significantly higher than scores obtained from a sample of volunteer medical students working in a state registration and reception center (t(62) = 1.225, *p* = 0.224) (Kindermann et al., unpublished data). However, according to other studies, STS is not uncommon in many groups of professionals working in different contexts with asylum seekers and refugees [19,20]. This apparently contradictory observation might be due to the limited time medical students spent in the reception center. There seems to be a dosis-effect relation concerning secondary traumatization which is referred to as the “building block effect” in studies on PTSD [64]. Nevertheless, some of our study’s participants mentioned that their cognitive schemas regarding themselves or the world in general had changed after they had completed their medical clerkship at the reception center. These changes mainly concerned a “disenchanted” view on the world as regards inequality, violence, and flight as a global issue as well as the resulting feeling of powerlessness. Taken together, these changes in cognitive schemas could be a sign for VT in some of the students. As a consequence, there should be an ongoing supervision for students who do a medical clerkship in these kinds of settings. Here, students with signs of psychological distress could be identified at an early stage and could receive psychological support, if necessary.

Concerning symptoms of depression, anxiety, and physical and mental wellbeing, our results did not reveal an increase in symptoms of distress after the students had completed their medical clerkship. One reason could be that it was optional for the students to do part of their medical clerkship in a state registration and reception center. The students could easily have conducted their placement in an “usual” inpatient clinic. Therefore, we can assume that there is a certain selection bias: presumably only students with a general interest in and an affinity to issues of global health and medical supply for refugees decided to carry out their medical clerkship at the reception center. Furthermore, it may be less likely that medical students who suffer from psychological burden or are prone to worry easily about getting in contact with contagious diseases or psychological traumatization would choose to do their internship in such a setting. Furthermore, we did not find significant differences when comparing the post PHQ-9 scores and the post GAD-7 scores to representative norm samples (t(2086) = 1.1135, *p* = 0.266; t(5050) = 0.3437, *p* = 0.731) [65,66]. However, we discovered that the medical students in the present sample had significantly lower depression and anxiety scores than a sample of first-semester medical students from a previous study by Bugaj et al. (t(310) = 3.8035, *p* < 0.0002; t(309) = 2.897, *p* = 0.004) [57]. In addition, the post-score for mental health was significantly higher than the score for mental health of the sample of first-semester students in the study by Bugaj et al. (t(310) = 3.424, *p* < 0.0001) [57]. We did not find any significant difference in the scores for physical health when comparing the two samples (t(310) = 0.723, *p* = 0.470).

### 4.2. Cultural Horizons and Sensitivity

In the post-interviews, the participating students repeatedly stated that they had obtained a better understanding of specific cultural characteristics and intercultural differences. With this ascertainment, the students not only alluded to communicative difficulties, i.e., when performing an anamnesis with refugees, trying to understand their complaints and symptoms, or asking them about their personal background stories. The students also referred to differences regarding gender or behavioral aspects which had to be taken into account. The participants considered this broadening of their personal and cultural horizon to be a valuable experience. In addition, the students noticed that disease, e.g., psychiatric disorders, also have a cultural component. More specifically, they discovered that there were different perceptions of psychological strain. This may become even more relevant for future doctors, as psychological problems seem to be more stigmatizing or shame-evoking in other cultures compared to Western countries [67]. Other research studies have discovered that people living in collectivistic societies may express psychological strain or traumatization more often as bodily complaints, for instance as pain or dizziness [68], instead of developing the “typical” Western symptomatology of PTSD or depression [69,70]. When conducting psychosocial anamnesis, the students therefore had to take into account psychological *as well as* somatic symptoms as a potential result of psychological traumatization. This indicates how important it is for future generations of doctors to be aware and sensitive of cultural differences because there will be an increase of globalization in the medical field [71].

### 4.3. Teachable Moments and Learning Achievements

Analyzing the students’ descriptions with the CanMEDS Role Model [42], we found that most of the teachable moments could be assigned to the students´ roles of “scholars” or “communicators”. This result could be affected by the special working context at the state registration and reception center. On the one hand, the medical students were often confronted with “exotic” diseases or diseases with a low prevalence in Western countries, for instance tuberculosis or malaria. Thus, the participants rarely had any professional experience in diagnosing or treating these diseases, apart from remembering some facts from the lecture. Because of this novel learning context, the students were likely to conceive themselves to be scholars. On the other hand, some basic administrative or communicative procedures at the outpatient clinic at the state registration and reception center are slower or even uncoordinated, e.g., due to the large number of refugees seeking treatment or because of language barriers and shortage of interpreters. Thus, the students often were in charge of coordinating and organizing various procedures for the refugees. Consequently, the students found themselves in the role of communicators.

Following the analysis of the reflective diaries applying Bloom´s taxonomy [41], the learning gain according to the reported teachable moments most frequently reached the stage of “understanding”. This could also be explained by the fact that students encountered a variety of rather rare diseases at the outpatient clinic. However, during their medical clerkship, the students began to recognize familiar conditions and were able to improve their current knowledge. This resulted in Bloom´s taxonomic stages of “analyzing” and “evaluating”. Based on the evaluation of both the interviews and the reflective diaries, we can assume that the participants had a major learning gain by completing their medical clerkship in the outpatient clinic of a reception center. These findings are in accordance with previous studies, supporting the view that medical students working in the context of medical care for asylum seekers do not suffer from significant psychological distress afterwards. Instead, they report on gaining a lot of medical and interactional knowledge and skills which eventually broaden their personal horizons [13,72]. Thus, conducting a medical clerkship in a state registration and reception center represents a significant extension of the medical curriculum: issues of global health are combined with the healthcare for asylum seekers. On the contrary, the introduction of medical students to the state registration and reception center has enabled a large number of organizational procedures to run much more smoothly. This also led to improved patient care, as students often helped the refugees with outpatient or bureaucratic matters. Furthermore, doctors in charge often felt a relief of their workload as the students performed basic routine activities, such as taking blood samples or measuring vital parameters. In short, the common interest in a common concern was both beneficial and motivating for doctors and students alike.

Against the background of a progressing globalization already impacting the medical field, we are convinced that medical curricula should include courses and internships in global health, healthcare for refugees, and cultural sensitivity. However, in the present study, we found some signs for negative changes in cognitive schemas in some of the students. Therefore, medical clerkships in state registration and reception centers should be preceded by introductory courses in order to provide knowledge to the medical students concerning (a) organizational procedures, (b) aspects of flight and forced migration, (c) the asylum procedure in Germany, (d) frequent physical disorders of refugees, (e) frequent mental disorders of refugees, and (f) intercultural communication. As mentioned above, such introductory courses can prepare the students for their assignments and reduce their worries or apprehensions regarding the practical encounter of refugees in a medical context. In addition, students could get information on the risk of suffering from psychological distress during or after their placement. Students doing longer assignments should be offered frequent supervision by a trained psychotherapist to ensure that psychological distress can be identified and treated at an early stage. Such supervision groups should take place once weekly for one hour and the group size should not exceed 6–8 students per session. If one would take the *n* = 22 participants of this study as an example, about three groups with one psychotherapist each would be needed.

### 4.4. Limitations

Several limitations of this study should be mentioned. First, our study was limited by the relatively small number of participants. Nevertheless, compared to the existing literature, this investigation constitutes the largest study sample in a study focusing on students and their learning achievements in the context of a medical clerkship at a state registration and reception center for forced migrants. Moreover, in contrast to studies that concentrate on similar research questions, we had a very high response rate to questionnaires. This leads to an acceptable comparability of our findings. A further limitation of the study was that the statistical analysis of the psychometric questionnaires in pre–post comparison was underpowered and that there was no control group. Lastly, the generalizability of our results may be restricted by the fact that it was not obligatory for students to do a medical clerkship in the state registration and reception center.

## 5. Conclusions

By conducting a medical clerkship at the outpatient clinic of a state registration and reception center, the students were able to develop awareness and sensitivity towards cultural differences and specificities. Cultural sensitivity played a pivotal role during the anamnesis and diagnostics of mental and physical conditions, as well as in general interactions with the refugees. The students reported a major gain in medical knowledge due to the encountering of rare physical diseases and psychological traumatization. Participants, moreover, indicated that they had improved their interactional, communicative, and organizational skills. The medical students did not display signs of significant psychological distress or STS as a result of their placement at the reception center. However, some of them showed signs for negative changes in cognitive schemas, which could be an indication for VT. To meet the demands of a progressing “medical globalization”, we are in favor of increasingly including theoretical and practical courses on global health and health care for asylum seekers in medical curricula. We suggest introductory courses and optional supervision for students in order to prevent negative effects, such as psychological distress.

## Figures and Tables

**Table 1:** Sociodemographic characteristics of the assessed *n* = 22 medical students.

**Measure**	
**Age (M ± SD)**	24.9 ± 2.6
**Gender**	
Female	11 (50.0%)
Male	11 (50.0%)
**Number of semesters already completed (M ± SD)**	9.1 ± 2.0
**Nationality**	
Germany	18 (81.8%)
Turkey	2 (9.1%)
Russia	1 (4.5%)
Austria	1 (4.5%)
**Prior medical clerkships**	17 (77.3%)
**Medical specialization of prior medical clerkships (multiple answers possible)**	
Surgery	11 (64.7%)
Radiology	7 (41.2%)
Psychiatry	6 (35.3%)
Internal Medicine	5 (29.4%)
**Prior education**	7 (31.8%)
**Special field of prior education (multiple answers possible)**	
Nursing	2 (28.6%)
Emergency rescue service	2 (28.6%)
Molecular biology	1 (14.3%)
Chemistry	1 (14.3%)
Dentistry	1 (14.3%)
**Aspired medical profession (multiple answers possible)**	
Psychiatry	6 (27.2%)
Neurology	5 (22.7%)
Pediatrics	4 (18.2%)
Surgery	3 (13.6%)
Oncology	3 (13.6%)
**Prior voluntary service**	17 (77.3%)
**Prior journeys to developing countries**	11 (50.0%)

**Table 2:** Results of the students’ reflective diaries: medical field of learning highlights, age, gender, and nationalities of patients and the students’ individual involvement during the consultation are given in numbers and percentages.

Measure	
**Learning highlights**	**125 (Missing: 54)**
General medicine	106 (84.8%)
Psychosocial medicine	9 (7.2%)
Pediatrics	5 (4.0%)
Gynecology	5 (4.0%)
**Age of patient (M ± SD)**	31.4 (±11,3)
**Patient’s gender**	121 (Missing: 58)
Female Male	43 (35.5%) 78 (64.5%)
**Patient’s nationality**	109 (Missing: 70)
Nigeria	23 (21.1%)
The Gambia	19 (17.4%)
Syria	10 (9.1%)
Somalia	7 (6.4%)
Eritrea	6 (5.5%)
Albania	6 (5.5%)
Georgia	5 (4.5%)
Macedonia	4 (3.6%)
Algeria	3 (2.7%)
Iraq	3 (2.7%)
Russia	3 (2.7%)
India	3 (2.7%)
Afghanistan	2 (1.8%)
Tunisia	2 (1.8%)
Turkey	2 (1.8%)
Serbia	2 (1.8%)
Togo	2 (1.8%)
Guinea	2 (1.8%)
Armenia	1 (0.9%)
Bosnia	1 (0.9%)
Iran	1 (0.9%)
Cameroon	1 (0.9%)
Kenya	1 (0.9%)
**Student´s function** (Multiple answers possible)	124 (Missing: 55)
Observing	55 (44.4%)
Assisting	67 (54.0%)
Independent work	33 (26.6%)
**Student’s practical involvement** (Multiple answers possible)	117 (Missing: 62)
Communicating	92 (78.6%)
Hands-on	55 (47.0%)

**Table 3:** Results from the psychometric questionnaires and group comparison with (A) volunteer medical students working in a state registration and reception center (Kindermann et al. unpublished data) and (B) first-semester medical students assessed in a previous study of Bugaj et al. [57].

Psychological Assessment—Descriptive Statistics and Comparison of T2 with Norm Sample							
Instrument	T1	T2	Norm Sample		Significance		
	Mean (SD)	Mean (SD)	Mean (SD)	*n*	*t*	df	*p*
FST [31–155]	-	40.81 (6.31)	38.52 (7.91) ^A^	62	1.225	62	0.224
PHQ-9 [0–21]	2.31 (3.14)	2.59 (1.97)	6.03 (4.19) ^B^	290	3.804	310	<0.001
GAD-7 [0–21]	2.77 (2.00)	2.70 (1.73)	5.34 (4.24) ^B^	290	2.897	309	0.004
SF-12 [0–100]							
Physical health score	56.45 (3.07)	55.85 (2.32)	55.06 (5.08) ^B^	290	0.723	310	0.470
Mental health score	50.88 (8.78)	52.94 (4.78)	45.33 (10.33) ^B^	290	3.424	310	<0.001

## Appendix A

**Table A1:** Quotations from the pre-interviews assigned to two different categories.

Quotations from pre-interviews
**Category (A) Expectations of the medical clerkship (110 codes)** I think when you are working in Psychosomatics (psychosocial outpatient clinic), you will witness a lot of things that will keep you occupied, but I do not think that you will suffer from sleepless nights later on. (A1.8) I think it (the medical clerkship) can be stressful when you are confronted with different fates and get in touch with the individual patients. (A1.2) Probably similar to other medical students, I am worried that my medical skills are not sufficient and that I will make mistakes. (A1.16) Actually, I do not have any specific fears (...), but I am worried about diseases which are not yet diagnosed, like tuberculosis; in my generation, I am not vaccinated anymore, and I do not know whether I could get infected. (A1.11) I feel respect for the cultural differences; (I ask myself) in which way will they (the refugees) handle our way of giving medical treatment? (A1.4) (I have been thinking about) the language barrier and how it will influence the quality of treatment. Otherwise I am just a little curious if there will be cultural difficulties and if you will try and speak about specific issues, or if you just formulate some issues badly. (A1.11) I think that it (the medical clerkship) will help to better understand other people or to learn to reflect before judging someone. (A1.9)
**Category (B) Motivations for the assignment (75 codes)** I look forward to perhaps broadening my horizon and to interacting with different patients; I can probably use this expertise for my future profession. (A1.9) (I am curious about) what kind of people I will meet and maybe about what their stories are and what kind of diseases they suffer from. (A1.17) (I am motivated) by my presumption that I can learn more things and make a greater contribution (in the reception center) than in a ‘normal’ general practice. (A1.1) I would like to get to know the people (refugees): what kind of people they are; maybe the spectrum of diseases or problems they bring along. Furthermore, I am interested in finding out in which way the flight has influenced or shaped people. (A1.1) I want to talk to the people (refugees) and listen to their stories. (A1.17) I find it interesting, because you get acquainted with extremely different people with different diseases. (A1.9)

**Table A2:** Quotations from the post-interviews assigned to two different categories.

Quotations from post-interviews
**Category (C) Subjective experiences during the medical clerkship (209 codes)** Usually, I was satisfied at the end of the day; working there seemed to be meaningful, I learned a lot, it was great. (A1.11) (Beforehand,) I worried about having to do a lot of administrative tasks there (in the outpatient clinic) but in fact I interacted directly with the patients all day long; I had the opportunity to do a lot of things on my own and to practice basic physical examinations; so, I did the standard examinations to find a diagnosis; this was really interesting. (A1.14) Well, I had the opportunity to do the anamnesis and the physical examination on my own. Therefore, I had to be aware of what I was doing, and I was supposed to develop an individual plan for the further diagnostic or therapeutic steps. (A1.10) In fact, the main problem was the language barrier, which was not really unbreachable but nevertheless a major obstacle, especially when there was no interpreter available. Some common foreign languages were helpful, for instance English or French. Fortunately, I speak a bit of French. (A1.2) A lot of people, especially refugees from Africa, reported complaints of the stomach and sometimes you do not really know where they (the complaints) come from. We discussed different causes. For instance, whether they (the complaints) were culturally conditioned or that some people just project psychological strain into their stomach more often. I often noticed that people came with unexplainable, long lasting stomach aches. Aside from that, they often had subconscious psychological problems, probably caused by their experiences, their flight, and the current situation in the refugee camp. (A1.7) It was always difficult, if they (the refugees) had been tortured before. It was difficult even if you transferred them to a psychologist because you cannot actually heal their condition and you do not know if they (the refugees) are able to talk about it (their experiences) in psychotherapy and if it (the therapy) will be successful then. It was also difficult when refugees had injuries for which treatment was not urgent, not an emergency. But they (the refugees) sometimes suffered from gunshot lesions to the bones. You did not do much in response because it was no acute (injury). (A1.9) In fact, it really made me sad and evoked consternation to see what other people have to go through right now in this moment, for instance the old couple; and that I myself am truly privileged; in comparison, my own problems appear to be much less important. (A1.7) There were a few individual background stories about which I had to think again, because those stories were just mad; although you hear about those stories in the newscast, it is crazy if someone who experienced such things on their own is standing right in front of you. Especially in the psychosocial outpatient clinic you hear a lot of those stories. In general medicine you sometimes do not even get in touch with it (the stories), but in Psychosomatics (the psychosocial outpatient clinic) there were some cases which kept coming back to me later on. (A1.4) As I expected, there were some different medical conditions compared to a ‘normal’ general practice (in Germany). For instance, hepatitis, sexually transmitted diseases, or other diseases which you are not likely to see in Germany. It was very interesting to encounter them (the diseases) here (in the outpatient clinic). (A1.14) (I found it interesting) to see medical conditions, which you normally do not encounter otherwise, like scabies or those tropical diseases; I sometimes wonder what happened to the patients after we transferred them to the specialists of the department of tropical medicine. (A1.9)
**Category (D) Subjective experiences after the medical clerkship and reflection (149 codes)** In fact, I had the feeling that this problem (war and flight) was so big, that you cannot do a lot about it. It feels like waking up, saying ´Okay, this is a huge problem, and I have to do something, but I am just not in the position to do so´. In fact, this is a sad feeling; but actually, it seems to be a mixture of positive and negative feelings. (A1.6) (I was affected by) the patients who had experienced terrible things on their flight or in their home countries. Yesterday, for example, I performed an ultrasound on a (female) patient, finding that she was pregnant; she was raped on her flight and this is where the child came from. (A1.4) I was definitely concerned by the radical inequality; that people in the world do not have the same chances at the start (of life); some people who were born here, like me, have good chances to achieve anything they want, whereas others have great difficulties to achieve what they want. In fact, for me this is not a novel thought, I have often experienced this in my life; but perceiving it (this inequality) in such an intensive way, like in the presence of the refugees, really affected me deeply. (A1.3) I think I have better insight now and can differentiate more clearly, who these refugees are, from which countries they originate, and what kind of problems they have to deal with. I think prior to that (medical clerkship), you merely had this vague notion of ´refugees´; now you are aware of what kind of people they are, how they came here, what they bring along with them. Now you can see them as human beings. (A1.5) In the context of learning, it (a medical clerkship in a reception center) makes more sense than being an intern in a general practice, where there are almost exclusively German patients and all cases are probably quite similar; so, it (the medical clerkship in the reception center) was very helpful for my medical studies. (A1.12) I found it quite informative to encounter those patients face-to-face; those patients are rather different, suffering from other conditions and conditions in other stages, because they did not have any treatment in their home countries; I found it quite interesting. (A1.13) Usually, on a normal day, I was heading back home and felt content; it (the medical clerkship in the reception center) felt very meaningful; I learned a lot, it was great. (A1.11)
